# Theoretical Model for the Prediction of Water Flux during the Concentration of an Olive Mill Wastewater Model Solution by Means of Forward Osmosis

**DOI:** 10.3390/membranes13080745

**Published:** 2023-08-21

**Authors:** Magdalena Cifuentes-Cabezas, Silvia Álvarez-Blanco, José Antonio Mendoza-Roca, María Cinta Vincent-Vela, José M. Gozálvez-Zafrilla

**Affiliations:** 1 Institute for Industrial, Radiophysical and Environmental Safety (ISIRYM), Universitat Politècnica de Valencia, Camino de Vera s/n, 46022 Valencia, Spain; magcica@upv.es (M.C.-C.); sialvare@iqn.upv.es (S.Á.-B.);; 2 Department of Chemical and Nuclear Engineering, Universitat Politècnica de València, Camino de Vera s/n, 46022 Valencia, Spain

**Keywords:** forward osmosis, modeling, tyrosol, olive mill wastewater, simulation

## Abstract

Currently, understanding the dynamics of the interaction between the agents in a process is one of the most important factors regarding its operation and design. Membrane processes for industrial wastewater management are not strangers to this topic. One such example is the concentration of compounds with high added value, such as the phenolic compounds present in olive mill wastewater (OMW). This process is a viable option, thanks to the forward osmosis (FO) process, osmotically driven by a saline stream. In this context, the transport of the solute and the solvent through the FO membranes, although essential to the process, remains problematic. This paper presents a study to predict, by means of a theoretical model, the water flux for two membranes (a cellulose triacetate flat sheet and a polyamide hollow fiber with integrated aquaporin proteins) with different characteristics using a sodium chloride solution as the draw solution (DS). The novelty of this model is the consideration of the contribution of organic compounds (in addition to the inorganic salts) to the osmotic pressure in the feed side. Moreover, the geometry of the modules and the characteristics of the membranes were also considered. The model was developed with the ability to run under different conditions, with or without tyrosol (the compound chosen as representative of OMW phenolic compounds) in the feed solution (FS), and was fitted and evaluated using experimental data. The results presented a variability in the model prediction, which was a function of both the membrane used and the FS and DS, with a greater influence of tyrosol observed on the permeate flux in the flat cellulose triacetate membrane.

## 1. Introduction

Currently, industries are increasingly interested in applying technologies to treat their wastewater. This is due to the great scarcity of water in some areas, as well as the rigorous laws regarding discharges [1]. Specifically, in the Mediterranean area, olive oil production is one of the main agricultural activities. This industry generates a large amount of olive mill wastewater (OMW), with a high organic content and the presence of phytotoxic compounds [2]. Although the proposed treatment strategies are usually successful in recycling water and reducing the amount of wastewater, most treatment technologies consume large amounts of energy. Therefore, it is necessary to develop treatment techniques that manage to provide treatment in an energy-efficient manner. The recovery of compounds present in wastewater begins as a strategy to amortize wastewater treatment, since it is possible to obtain products with high profit from waste, promoting a circular economy [3]. In OMW, the phenolic compounds mainly provide its phytotoxic character. These compounds, besides being recalcitrant, possess outstanding antioxidant properties. Among the phenolic compounds present in OMW, tyrosol is one of the major compounds, being recognized for its antioxidant, anti-inflammatory, and antimicrobial properties. Therefore, its recovery is of great interest for its future commercialization in cosmetic, food, and pharmaceutical industries [4].

Concentration processes, besides being part of the main operating units of many industrial sectors, either for the processing, obtaining, and/or extraction of products, have also been studied regarding the recovery of compounds and the treatment of wastewater. Among these, pressure-driven membrane processes are widely used in various applications. Although these processes offer several advantages over traditional technologies, such not requiring additives, to date, there is still no technology that offers a fully satisfactory solution that allows treatment with low energy cost [5]. Within membrane processes, forward osmosis (FO) has recently received a great deal of interest in wastewater treatment. This is due to the fact that, compared to membrane pressure processes, it exhibits a lower fouling potential and low energy consumption, allows for the simultaneous treatment of two currents in a single treatment step, and also enables the treatment of liquids that are not suitable for other membrane processes [6]. These characteristics of the FO process make it stand out from other phenolic compound concentration/recovery processes. Although good results have been obtained using direct contact membrane distillation (DCMD), in DCMD, it is necessary to maintain a ΔT = 30 °C between the FS and the permeate, making the process more expensive [7], while the phenolic compounds may also be affected by the increase in temperature.

In FO, it is the concentration gradient (also called osmotic pressure difference, ∆π) that acts as the driving force to generate a flow of water through the membrane, which requires little external energy. The process separates two streams, one with low osmotic pressure (feed solution, FS) and other with higher osmotic pressure (draw solution, DS), through a semi-permeable membrane. Due to the difference in osmotic pressure between both streams, water permeates from the FS side to the DS side of the membrane, thus concentrating the FS while simultaneously diluting DS [8]. In the case of OMW, the phenolic compounds are concentrated in this manner for subsequent recovery.

However, this FO process also presents some obstacles, such as reverse solute flux and concentration polarization (CP), leading to a decrease in FO efficiency. CP occurs near the membrane walls in FS and DS—a phenomenon called external concentration polarization (ECP). Nevertheless, it is a relatively less significant problem than internal concentration polarization (ICP) in the FO processes. ICP in the support layer represents a significant obstacle to the permeation of water through the membrane. The extracting solute penetrates the porous support layer of the membrane according to Fick’s law. The diffused solute is then diluted with the water diffused from the FS to the support layer, generating a reduction in the osmotic pressure gradient across the active layer and therefore, a corresponding reduction in water flux [9,10]. However, as this is inherent to the membrane structure, result is difficult to mitigate [11].

On the other hand, more factors can influence the performance of the FO process, including membrane-specific factors (material, structure, surface area, and configuration), FS and DS characteristics, and operating parameters [12]. Therefore, as indicated by Singh et al. [13], understanding the dynamics of solute and solvent transport through osmotic-driven membranes, although essential, remains an unsolved problem at this time.

Different models have been developed to explain the fundamental process of FO, such as the early models of Lee et al. [14] and Loeb et al. [15], which considered a reverse solute flow, and this the version was later improved by Tang et al. [16], who including the concept of inverse solute selectivity, or the model of Suh and Lee [9], which considered ECP. However, although there has been further development and research regarding the FO process in recent years, there is still a scarcity of literature concerning modeling studies of the FO process. Some attempts have been made in regards to the treatment of phenolic compounds using FO [17,18]. To our knowledge, to date, there are no studies prior to this work considering FO modeling with OMW, with either real or simulated solutions.

The first models developed for the prediction of permeate flux in these processes were proposed by Ref. [14]. Although these models were the basis for understanding the FO process, they were developed for RO and PRO processes (reverse osmosis and pressure retarded osmosis). Then, it was the authors of [15] who transformed these models for use in FO. These authors were able to predict both the permeate flux and the reverse passage of the salts under a theoretical model that is still widely used today. However, these models are quite generic for analyzing the differences between the impact generated by both the salts in the draw solution and the components that are present in the feed. That is why these models, in spite of being the basis for understanding the FO process, must be modified and improved, according to the specific work characteristics. More recently, Ref. [19] performed additional studies regarding ICP (internal concentration polarization). However, as The authors of [20] point out in their work on the model prediction of flux behavior considering ECP (external concentration polarization) and ICP, at high DS concentrations (greater than 1.0 M NaCl) and high water fluxes, some theoretical models overestimate the flux of water across the membrane at the corresponding osmotic pressure. Therefore, they emphasize the need for a more accurate model for flux prediction, process optimization, fouling studies, treatment of potential feed streams, etc.

It is also very relevant to analyze different types of FS, considering that in the aforementioned studies [15,19], the feed and draw solutions used were deionized water and NaCl solutions. The NaCl concentrations varied from 0 (deionized water) to 1.0 M for FS and from 0.05 to 1.5 M for DS. Thus, more specific models must be developed. There are some considerations that could be taken into account for a best prediction of water flux in FO processes. Thus, Haupt et al. [21] analyzed three theoretical models for the modeling of the FO process for different types of wastewater from the automobile industry (cathodic dip painting rinsing water, cathodic dip painting wastewater, paint shop pre-treatment wastewater, and cooling tower circulation water). They concluded that, in some experiments, the permeate fluxes could be fitted to those predicted by the models, but in others, the water fluxes were overestimated or underestimated. They attributed this to the fact that fouling is not considered in the models, as well as to the complex composition structure of industrial wastewater, which may also influence the FO process. It is also important to note that, as the concentration process progresses, the effect of the composition of the feed on the driving force becomes more relevant. Some compounds show much greater rejection than others. These aspects are very important in a field such as the treatment of OMW in which different compounds are involved.

The aim of this study was to predict, by means of a theoretical model, the water flux of two membranes of different characteristics: a hollow fiber membrane of thin-film composite polyamide (TFC), integrating aquaporin proteins, and a flat-sheet membrane of triacetate of cellulose (CTA). This model have the ability to perform under different conditions, with or without tyrosol (the target phenolic compound of OMW) in the FS. The mathematical model takes into account mass balance, as well as membrane characteristics and concentration polarization equations. The model was fitted to and evaluated with data from a dynamic FO process. Two types of characterization tests were carried out, depending on the composition of the FS: (*i*) sodium chloride solutions and (*ii*) OMW model solutions (sodium chloride and tyrosol).

## 2. Theory

In FO, permeation is mainly influenced by the species making the greatest contribution to the chemical potential and consequently, to the osmotic pressure, such as low molecular weight salts. In the dense layer of an FO membrane, water flux is driven by the osmotic pressure difference associated with the solute concentrations in the FS and DS. For ideal conditions, the water flux (*J_w_*) in an FO process is as follows [14,19]:

(1) $$J_{w}=A_{w}\cdot \sigma \cdot \left(\pi_{D}-\pi_{F}\right)$$

where *A_w_* is the membrane permeability coefficient, *σ* the reflection coefficient, and *π_D_* − π*_F_* is the difference in osmotic pressure between the feed and draw solutions across the membrane selective layer. Assuming a perfect barrier, i.e., the salt does not cross the membrane, the reflection coefficient can be considered as equal to one.

External concentration polarization, defined as the increase or decrease in the solute concentration near the membrane surface with respect to the bulk solution, is common in any FO process and must be taken into account. However, most of the commercially available membranes are asymmetric, with a dense active membrane layer supported on a porous layer. In this case, internal concentration polarization, produced on the membrane support layer, can affect FO performance more significantly than ECP due to reverse salt diffusion from the DS towards the FS.

### 2.1. Transport Model for Asymmetric FO Membranes

**Figure 1:**
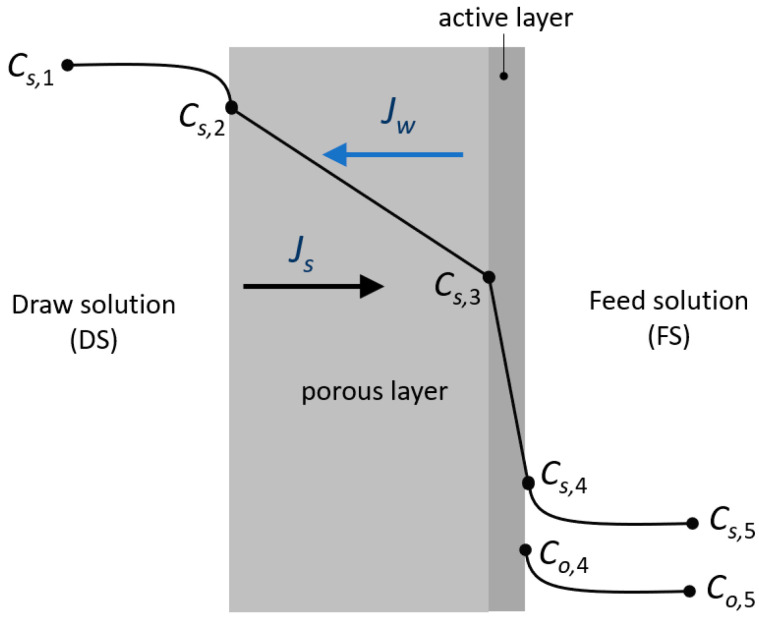
Concentration profiles across the membrane in forward osmosis mode for salt (*s*) and phenolic organic compounds (*o*).

Some studies neglect the salt concentration of the feed solution, but in this study, as in Refs. [14,19], it is considered. However, a novelty of our study is the consideration of the contribution to the osmotic pressure on the feed side of the organic compounds, in addition to that of the salt. The situation studied is shown in Figure 1, which shows the salt concentration profile through an asymmetric membrane, with a porous support layer and a dense active layer working in forward osmosis mode.

According to film theory [20], the steady state concentrations in the bulk feed solution and at the membrane wall are related to the water flux and the transport coefficient of the components in each compartment:

(2) $$J_{w}=-k_{sD}\cdot \ln\frac{C_{s,1}}{C_{s,2}}=-k_{sF}\cdot \ln\frac{C_{s,4}}{C_{s,5}}=-k_{oF}\cdot \ln\frac{C_{o,4}}{C_{o,5}}$$

The mass transport coefficients, *k_i_*_,*j*_ in Equation (3), depend on the hydrodynamics of the draw (D) or feed (F) compartments and the solute characteristics. For a rectangular channel and turbulent flow, they can be obtained from a Sherwood (*Sh*) correlation (Equations (3) and (4)) taking into account the Reynolds (*Re*) and Schmidt (*Sc*) numbers [22]:

(3) $$k=\frac{Sh\cdot D}{d_{h}}\;$$

(4) $$Sh=0.04\cdot Re^{0.75}\cdot Sc^{0.33}$$

where *D* is the solute diffusivity in the solvent and *d_h_* the hydraulic diameter of the membrane feed channel. The reverse salt flux (*J_s_*) through the active layer of the membrane is given by the following equation:

(5) $$J_{s}=B_{s}\cdot \left(C_{s,3}-C_{s,4}\right)$$

where *B_s_* is the solute permeability coefficient, and *C_s_*_,3_ and *C_s_*_,4_ are the solute concentration in the draw and feed interfaces of the membrane active layer, respectively (see Figure 1).

The ICP profile in the porous layer is obtained using the convection–diffusion equation for the solute flux [15]:

(6) $$J_{s}={-D}_{s}\cdot \epsilon \cdot \frac{dC_{s}}{dx}-J_{w}{\;C}_{s}$$

The integration of Equation (6) in the porous layer between points 3 and 4 yields:

(7) $$J_{w}=\frac{1}{K_{s}}\ln\left(\frac{J_{w}\cdot C_{s,3}+B_{s}\cdot \left(C_{s,3}-C_{s,4}\right)}{J_{w}\cdot C_{s,4}+B_{s}\cdot \left(C_{s,3}-C_{s,4}\right)}\right)$$

where *K_s_* is the internal polarization modulus (Equation (8)), which depends on the diffusion coefficient of the salt (*D_s_*) and a structural membrane parameter *S* (Equation (9)) calculated from the thickness (∆*x*), tortuosity (*τ*) and porosity (*ε*) of the porous support layer.

(8) $$K_{s}=\frac{S}{D_{s}}\;$$

(9) $$S=\frac{\tau \cdot \Delta x}{\epsilon \cdot D_{s}}$$

At a given temperature, it was assumed that the contribution to the osmotic pressure of each component in a position *j* is proportional to the component concentration through a constant osmotic coefficient (*α*), taking into account the modified van ’t Hoff formula [23,24]:

(10) $$\pi_{j}=\alpha_{s,j}\cdot C_{s,j}+\alpha_{o,j}\cdot C_{o,j}$$

Equation (10) was applied, without taking into account the osmotic contribution of the organic compounds for points *j* = 1, 2, 3 in Figure 1, but for the feed side (point 4) and the feed bulk (point 5), the osmotic contribution of other components, different from salt, was not neglected.

Applying Equation (1) to both sides of the active layer:

(11) $$J_{w}=A_{w}\cdot \left(\pi_{3}-\pi_{4}\right)=A_{w}\cdot \left(\propto_{s}C_{s,3}-\pi_{4}\right)$$

where *C_s_*_,3_ can be expressed as:

(12) $$C_{s,3}=\frac{A_{w}\pi_{4}+J_{w}}{\propto_{s}A_{w}}$$

Therefore, the combination of the previous equations results in Equation (13), proposed for the numerical determination of the water flux, considering this osmotic contribution:

(13) $$J_{w}=\frac{1}{K_{s}}\ln\left(1-\frac{J_{w}\cdot (A_{w}\cdot \left(\pi_{4}-\alpha_{s}\;C_{s,2}\right)+J_{w})}{B_{s}\cdot \left(A_{w}\cdot \left(\pi_{4}-\alpha_{s}\;C_{s,4}\right)+J_{w}\right)+A_{w}\;\pi_{4}\cdot J_{w}+J_{w}^{2}}\right)$$

Once water flux is known, the salt flux can be calculated using:

(14) $$J_{s}=B_{s}\cdot \left(C_{s,3}-C_{s,4}\right)=B_{s}\cdot \left(\frac{A_{w}\cdot \pi_{4}+J_{w}}{\alpha_{s}\;A_{,w}}\right)-C_{s,4}$$

To consider the effect of ECP, Equations (13) and (14) must be combined with Equation (2) to obtain the osmotic pressure at point 4 (feed side) from the information for the respective bulk concentrations (points 1 and 5) and the specific transport coefficients:

(15) $$\pi_{4}=\pi_{Fs}\exp\left(\frac{J_{w}}{k_{Fs}}\right)+\pi_{Fc}\exp\left(\frac{J_{w}}{k_{Fc}}\right)$$

(16) $$\pi_{2}=\pi_{Ds}\exp\left(-\frac{J_{w}}{k_{Ds}}\right)$$

Note that, in the absence of ECP, if the osmotic pressure is due exclusively to salt, then Equation (13) reduces to the commonly used equation, which McCutcheon and Elimelech [19] used to describe the FO process:

(17) $$J_{w}=\frac{1}{K}\ln\left(\frac{A_{w}\;\pi_{2}+B_{s}}{A_{w}\;\pi_{4}+B_{s}+J_{w}}\right)$$

which can be used for initialization purposes during the parameter fitting.

### 2.2. Dynamic Model of the Forward Osmosis System

The FO system was modeled as two homogeneous compartments exchanging water and solutes across a membrane of area *A*. The osmotic pressure difference between the FS and DS is the driving force that causes the flow of water from the feed solution to the draw solution. Conversely, a small salt flux is received by the feed solution.

Assuming constant density, the balance equations for the feed and draw compartments, whose volume is represented by *V*, lead to the following ordinary differential equation system:

(18) $$\frac{dV_{D}}{dt}=J_{w}\cdot A$$

(19) $$\frac{dV_{F}}{dt}=-J_{w}\cdot A$$

(20) $$\frac{dC_{D,s}}{dt}=\left(-J_{s}-J_{w}\cdot C_{D,s}\right)\cdot \frac{A}{V_{D}}$$

(21) $$\frac{dC_{F,s}}{dt}=\left(-J_{s}-J_{w}\cdot C_{F.s}\right)\cdot \frac{A}{V_{F}}$$

(22) $$\frac{dC_{F,c}}{dt}=\left(J_{w}\cdot C_{F.c}\right)\cdot \frac{A}{V_{F}}$$

where *J_s_* and *J_w_* are functions of the composition in both compartments, calculated using the most accurate transport model given by Equations (10)–(17).

## 3. Materials and Methods

### 3.1. Feed and Draw Solutions

For the experimental tests, model solutions were used on both membrane sides. For the FS, a tyrosol solution of 1 g∙L^−1^ in distilled water was used. This concentration was selected, as it is similar to the total phenolic concentration measured in the characterization of an OMW obtained in a previous work [25]. Sodium chloride (VWR chemicals, Belgium), at concentrations of 30 g·L^−1^ and 200 g·L^−1^, was used for the DS. Both salt concentrations were selected because in other studies [26], they led to high permeate fluxes and low reverse salt fluxes. As for the 30 g·L^−1^ of NaCl concentration, this was selected based on the characterization of an actual brine obtained from the table olive producing industry [27].

### 3.2. Forward Osmosis Test

All the tests were carried out in FO in countercurrent flow, with the FS in front of the active layer of the membrane, due to its higher efficiency in terms of water flux and fouling [28]. Two different membranes were tested, the FTSH2O^TM^ flat sheet membrane (Fluid Technology Solutions, Albany, OR, USA), using the CFO42 module (Sterlitech Corporation, Auburn, WA, USA), and the HFFO2 hollow fiber membrane (Aquaporin Inside, Lyngby, Denmark), incorporated in its own module. The specific characteristics of each membrane are presented in Table 1. The fit of the proposed model was evaluated by comparing the simulated and experimental results. The effects of both concentrative ICP and dilutive ECP were investigated simultaneously.

First, a characterization of both membranes was carried out to obtain the specific parameters of each one (*A_w_*, *B_s_* and *K_s_*). For this, different DS concentrations were used, all prepared with NaCl, in quantities ranging between 25 and 200 g·L^−1^ (0.43 to 3.5 M, respectively), and pure water (conductivity < 40 µS·cm^−1^). The operating conditions for the characterization tests were the same for both membranes, with flow rates of 25 L·h^−1^ for the FS and 15 L·h^−1^ for the DS. Then, two tests were carried out to evaluate the prediction of the selected model: (*i*) a test using distilled water as the FS and a concentration of 200 g·L^−1^ of NaCl for the DS; and (*ii*) a test using 1 g·L^−1^ of tyrosol as the FS and 30 g·L^−1^ of NaCl as the DS (see [29] for the justification of the concentrations used). In the case of the FTSH2O membrane, the FS and the DS were pumped at a flow rate of 30 L·h^−1^, while for the HFFO2 membrane, the experiments were carried out at 60 L·h^−1^ for the FS and 25 L·h^−1^ for the DS (based on manufacturer recommendations and previous studies carried out by the research group). The salt concentration was measured by means of conductivity, as well as using Merk kits (Darmstadt, Germany) measuring the ion Cl^−^. For the tyrosol concentration measurement, the Folin–Ciocalteau spectrophotometric method [30] was employed, using tyrosol as a standard (Maybridge, Altrincham, UK).

The permeate flux *J_w_* was measured experimentally using the weight variation of the DS, as described in Equation (23), while the solute flux *J_s_* was determined by considering the salt mass variation in the FS using Equation (24).

(23) $$J_{w}=\frac{\Delta m}{A\cdot \Delta_{t}}$$

(24) $$J_{s}=\frac{V_{t}\cdot C_{t}-V_{t-1}\cdot C_{t-1}}{A\cdot \Delta_{t}}$$

In the above equations ∆*m* corresponds to the mass change of the draw solution, ∆*_t_* is the time interval between the mass measurements, and *C_t_* and *V_t_* are the salt concentration and the volume of the feed solution, respectively, at time *t*.

Therefore, to model the behavior of an FO membrane, there are three parameters that fully describe its performance: water permeability (*A_w_*), salt permeability (*B_s_*), and a structural parameter (*K_S_*), which is related to the internal polarization modulus *K_s_*.

## 4. Results and Discussion

### 4.1. Model Parameter Determination

The main objective of the characterization of the membrane is to determine the value of the internal polarization modulus K. It is a parameter that depends mainly on the nature of the solute and the porous medium through which it diffuses; it should, therefore, be relatively constant for a given solute and membrane, whatever the value of *J_w_* or ∆π [15].

**Figure 2:**
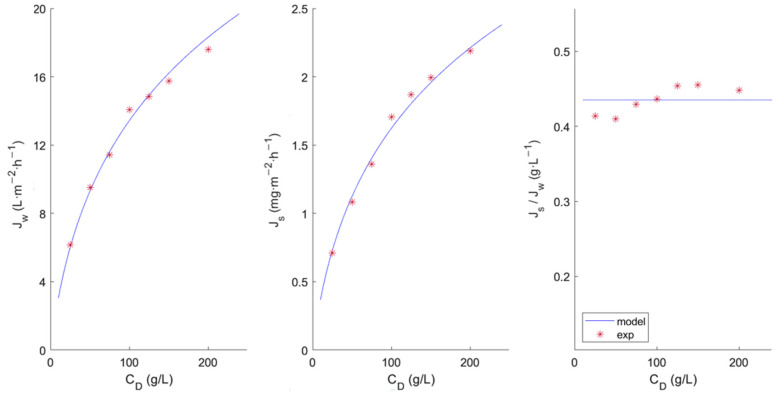
Characterization of the FTSH2O membrane by means of the water flux (*J_w_*) and reserve salt passage (*J_s_*) at different salt concentrations in the draw solution.

**Figure 3:**
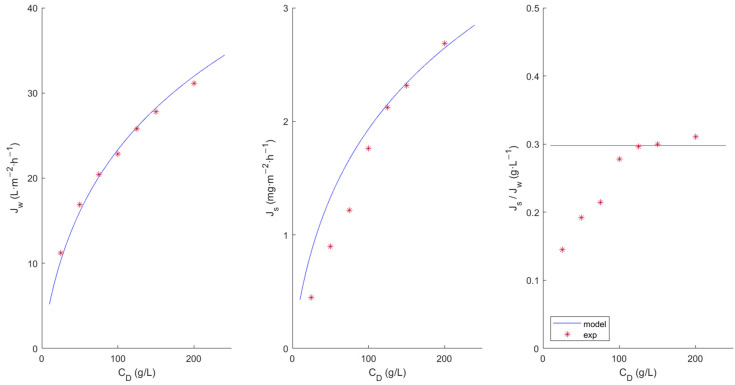
Characterization of the HFFO2 membrane by means of water flux (*J_w_*) and reserve salt passage (*J_s_*) at different salt concentrations in the draw solution.

Figure 2 and Figure 3 show the experimental data and the fit obtained with the model. It can be seen that permeate flux increases upwards along with the increase in the concentration of the DS solution. However, a pronounced non-linear trend is observed. McCutcheon and Elimelech [19] pointed out that a significant salt passage from the draw solution moves the representation of J_w_ versus the osmotic pressure difference away from linearity due to the presence of ICP. This may be due to the orientation of the membrane active layer (the FS faced the dense active layer), since in this orientation, the ICP effect predominates over the ECP effect. Therefore, a dilutive ECP and a concentrative ICP would result [13].

Regarding the proposed model, the good fit of the model can be clearly observed for both membranes, predicting water fluxes at various SD concentrations close to the experimental value. However for the HFFO2 membrane (Figure 3), it seems to predict a higher salt flux than that observed at lower concentrations. This could be due to the fact that the membranes did not reach a stationary state, which would justify the observed variation in *J_s_*/*J_w_*. Longer trials should be performed to study this phenomenon more precisely.

The results of *A_w_*, *B_s_*, and *K_s_* for the HFFO2 (HF) and FTSH2O (FT) membranes are shown in Table 2. They were obtained by minimizing the quadratic error for *J_w_* and *J_s_* in the characterization test. To determine the values of these parameters, an initial calculation was carried out, without considering CP. Then, the model was applied in the optimization procedure to obtain the model parameters for each membrane. Through adjustment, *A_w_* and *B_s_* arise naturally from the results of *J_w_* and *J_s_*; however *K_s_* is a parameter that cannot be directly measured in experimental tests, and it is dependent on the membrane structural parameter (*S*) defined in Equation (8).

The values of the permselective parameters obtained by fitting the model to the experimental data (Table 2) were compared to the values provided in the literature for each membrane. In the case of the FTSH2O membrane, the parameters *A_w_* and *B_s_* were 32.8% and 50.9% lower, respectively, than those obtained in Ref. [31] (*A_w_* = 1.92 × 10^−12^ m^2^·s^−1^·kg^−1^ and *B_s_* = 9.44 × 10^−8^ m·s^−1^). However, in the case of the HFFO2 membrane, the *A_w_* parameter was 50.4% lower, but *B_s_* was 20.9% higher, in comparison with the values obtained in Ref. [32] (*A_w_* = 4.427 × 10^−12^ m^2^·s^−1^·kg^−1^ and *B_s_* = 4.17 × 10^−8^ m·s^−1^). The differences in the parameter values were not very significant, but it was observed that the relationship between *A_w_* and *B_s_* changed in a different manner for each membrane, after fitting to the model proposed in this work. The difference could be due to the methodology used by the other authors to determine the parameters. The parameters for the FTSH2O membrane were determined following standard procedures [33,34], while for the HFFO2 membrane, the parameters were calculated using the modified model of Bui et al. [35] for randomly packed fiber bundles.

The use of different salts under the same operating conditions can generate different results for the parameters. Therefore, as Sanahuja-Embuena et al. [32] point out, the modeling method requires subjective judgment, and the calculated values should be treated as estimations.

### 4.2. Membrane Test and Model Predictions

**Figure 4:**
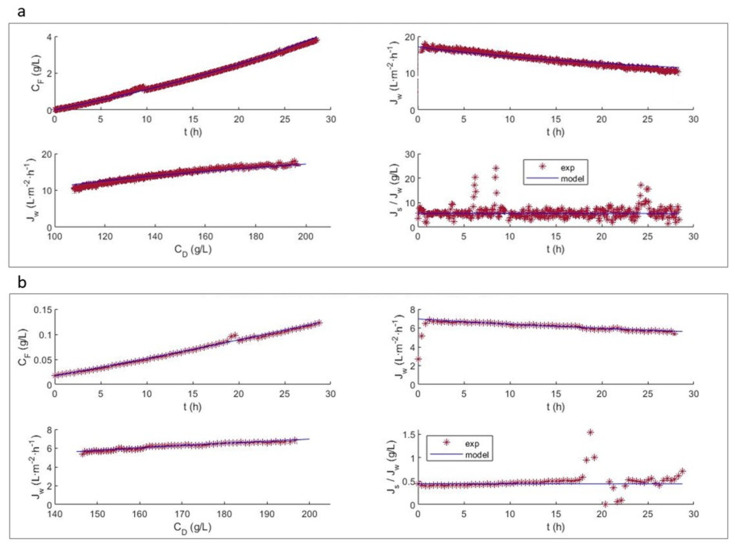
Comparison of experimental and predicted data for the FTSH2O membrane (**a**) test (i) with 200 g L^−1^ NaCl as the DS and distilled H_2_O as the FS; (**b**) test (ii) with 30 g L^−1^ NaCl as the DS and 1 g L^−1^ tyrosol as the FS.

**Figure 5:**
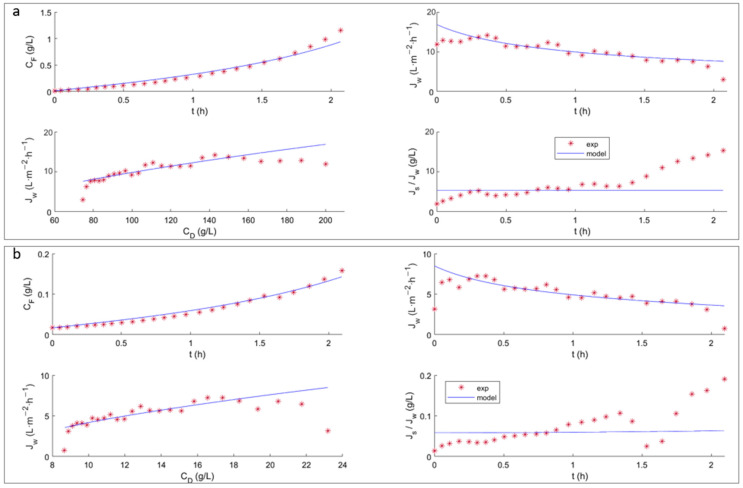
Comparison of experimental and predicted data for the HFFO2 membrane (**a**) test (i) with 200 g L^−1^ NaCl as the DS and distilled H_2_O as the FS; (**b**) test (ii) with 30 g L^−1^ NaCl as the DS and 1 g L^−1^ tyrosol as the FS. The parameters were readjusted with respect to the original test using salt.

Flux modeling is essential to predict how flux performance changes under varying system conditions or membrane structures. Using the model presented in Section 2, the results obtained with NaCl (200 g·L^−1^) as the DS and distilled H_2_O as the FS (Figure 4a and Figure 5a), and those with NaCl (30 g·L^−1^) as the DS and 1 g·L^−1^ tyrosol as the FS (Figure 4b and Figure 5b) are shown for the FTSH2O (Figure 4) and HFFO2 (Figure 5) membranes.

For the system modeling study, the results obtained are presented again, but this time, the fitting was performed using data regarding the evolution of the volumes of the DS and FS (*V_D_* and *V_F_*, respectively) and the DS and FS concentrations (*C_D_* and *C_F_*, respectively) in the tank. This type of adjustment can be more accurate, since the temporal derivatives of the fluxes are very sensitive to disturbances, while the fitting based on a cumulative variable dampens the results. Additionally, to compare the flux predicted by the model and the experimental results, a moving average smoothing function was used for the flux.

In general, it can be seen in both Figure 4 and Figure 5 that, after readjustment of the parameters, the volume, concentration and Jw trends are met. In both membranes, the values obtained for *A_w_* and *B_s_* were lower than those previously observed (Table 2). In addition, when tyrosol was present, these levels were even lower. This may be a consequence of the tyrosol creating an additional layer of resistance that hinders the passage of water and salt. A more detailed study using a bilayer model could be attempted.

When working with model solutions that only contain tyrosol, there is no influence of fouling, due to the deposit of other solutes on the membrane surface. In this case, the decrease in *J_w_* is mainly due to the decrease in the osmotic pressure over time, and is mainly due to dilutive ICP (a characteristic of asymmetric FO membranes) [36]. In other studies where a CTA membrane was used to treat OMW, ATR-FTIR analysis was used to characterize the membrane surface after the process. A characteristic peak around 1580 cm^−1^ was observed, which is attributable to the C–C stretching that occurs in the aromatic rings of polyphenols, demonstrating their adhesion to the surface of the membrane [37]. It is important to note that due to the almost total absence of hydraulic pressure in the FO operation, less compaction of the fouling layer occurs, thus facilitating the recovery of the membrane.

The effect of ECP and ICP on the determination of water flux can be observed. The reduction in flux is mainly attributed to the decrease in the osmotic pressure difference, with the impact of ICP and ECP not being significantly prominent for the test performed with distilled water as the FS (Figure 4a and Figure 5a). However, the impact of ECP and ICP is more complex in the FS containing more foulants such as tyrosol, and it increases under high osmotic conditions (Figure 4b and Figure 5b). It can also be observed that the flux of the FTSH2O membrane is more significantly influenced by both the difference in NaCl concentration in the DS and the presence of tyrosol in the FS, decreasing the initial flux by half. However, this was not observed with the HFFO2 membrane, which showed stable fluxes of similar values.

It was expected that, having obtained the parameters by testing in the absence of tyrosol, the model would better fit the results of test (i) compared to test (ii); however, this was not the case. It was observed that the parameters required significant readjustments for test (i) performed with the FTSH2O membrane (Figure 4a), even for the value of *K_s_*. This may be because the tests presented in the previous section (Figure 2) and the test using tyrosol (Figure 4b) were performed with a different membrane coupon. However, this did not occur with the HFFO2 membrane, despite the fact that, as in the previous test, the salt assays of test (i) were performed with a new piece of membrane. This would imply a higher variability in the flat membrane than in the hollow fiber example. This could also explain the discrepancies between the mean evolution slope and experimental slope observed in the Jw subfigures. This confirms the manufacturing variability of this type of membrane.

It can be seen that the experimental *J_s_*/*J_w_* value for the flat membrane (Figure 4) presents a deviation from linearity between 18 and 22 h of testing. This is due to a change in the conductivity meter used to adapt to the conductivity values reached. Despite the discontinuity observed, the model achieved a good representation of the information, without being affected by these anomalous data. For the HFFO2 membrane (Figure 5), it is again observed that the experimental *J_s_*/*J_w_* ratio increased with time, while according to the model, it should not vary as significantly and should be very similar to the *A_w_*/*B_s_* ratio. However, the ratio decreased with time and therefore, it also reduced along with the decrease in CD. This result therefore reinforces the previous hypothesis regarding a transient stabilization of the membrane behavior.

## 5. Conclusions

Most forward osmosis modeling approaches do not consider the concentration of components other than salt in the feed solution. It is even common not to consider the salt passage. In this work, salt passage and the effect of organics on osmotic pressure have been taken into account in the modeling, along with the existence of internal and external polarization layers.

The experimental results were obtained with a model solution that simulates olive mill wastewater, which included tyrosol as a solute. The experimental data was adjusted to the developed model, and it was possible to estimate the solvent and solute permeability parameters, as well as the polarization modulus, starting from values obtained in the literature and readjusting them.

The differences between the permselective parameters obtained after fitting the model to the experimental data and those found in the literature were not significant. However, for both membranes, the ratio between the solvent and the solute permeability coefficients was affected differently. The permeate flux levels predicted by the model were very close to the experimental values, especially in the case of the FTSH2O membrane.

In conclusion, the inclusion of the contribution of low molecular weight organic compounds to the osmotic pressure may contribute to a better estimation of the transport parameters of a forward osmosis process applied to real feed streams than estimations obtained using theoretical models that only consider the contribution of salts to osmotic pressure.

## Figures and Tables

**Table 1:** Characteristics and properties of the FO membranes.

Parameter	FTSH2O	HFFO2
Material	Cellulose triacetate	Polyamide with integrated aquaporin proteins
Configuration	Flat sheet	Hollow fiber
Area (m^2^)	0.0042	2.3

**Table 2:** Main parameters obtained by fitting the model to the experimental data.

Membrane	*A_w_* (m^2^·s·kg^−1^)	*B_s_* (m·s^−1^)	*K_s_* (s·m^−1^)
FTSH2O	1.29 × 10^−12^	4.68 × 10^−8^	2.88 × 10^5^
HFFO2	2.17 × 10^−12^	5.40 × 10^−8^	1.60 × 10^5^

## Data Availability

The raw data of this study are available on request.
